# Supporting families managing childhood eczema: developing and optimising eczema care online using qualitative research

**DOI:** 10.3399/BJGP.2021.0503

**Published:** 2022-05-17

**Authors:** Katy Sivyer, Emma Teasdale, Kate Greenwell, Mary Steele, Daniela Ghio, Matthew J Ridd, Amanda Roberts, Joanne R Chalmers, Sandra Lawton, Sinead M Langan, Fiona Cowdell, Emma Le Roux, Sylvia Wilczynska, Hywel C Williams, Kim S Thomas, Lucy Yardley, Miriam Santer, Ingrid Muller

**Affiliations:** Department of Psychology, University of Southampton, Southampton; lecturer, University of Portsmouth, Portsmouth.; University of Southampton, Southampton.; University of Southampton, Southampton.; University of Southampton, Southampton.; University of Manchester, Manchester; research fellow, University of Southampton, Southampton.; University of Bristol, Bristol.; Nottingham.; University of Nottingham, Nottingham.; Rotherham NHS Foundation Trust, Rotherham.; London School of Hygiene and Tropical Medicine, London.; Birmingham City University, Birmingham.; University of Hertfordshire, Hertfordshire.; University of Southampton, Southampton.; University of Nottingham, Nottingham.; University of Nottingham, Nottingham.; University of Bristol, Bristol; professor of health psychology, University of Southampton, Southampton.; University of Southampton, Southampton.; University of Southampton, Southampton.

**Keywords:** atopic eczema, caregivers, family practice, internet-based intervention, paediatric dermatology, qualitative research

## Abstract

**Background:**

Childhood eczema is often poorly controlled owing to underuse of emollients and topical corticosteroids (TCS). Parents/carers report practical and psychosocial barriers to managing their child’s eczema, including child resistance. Online interventions could potentially support parents/carers; however, rigorous research developing such interventions has been limited.

**Aim:**

To develop an online behavioural intervention to help parents/carers manage and co-manage their child’s eczema.

**Design and setting:**

Intervention development using a theory-, evidence-, and person-based approach (PBA) with qualitative research.

**Method:**

A systematic review and qualitative synthesis of studies (*n* = 32) and interviews with parents/carers (*n* = 30) were used to identify barriers and facilitators to effective eczema management, and a prototype intervention was developed. Think-aloud interviews with parents/carers (*n* = 25) were then used to optimise the intervention to increase its acceptability and feasibility.

**Results:**

Qualitative research identified that parents/carers had concerns about using emollients and TCS, incomplete knowledge and skills around managing eczema, and reluctance to transitioning to co-managing eczema with their child. Think-aloud interviews highlighted that, while experienced parents/carers felt they knew how to manage eczema, some information about how to use treatments was still new. Techniques for addressing barriers included providing a rationale explaining how emollients and TCS work, demonstrating how to use treatments, and highlighting that the intervention provided new, up-to-date information.

**Conclusion:**

Parents/carers need support in effectively managing and co-managing their child’s eczema. The key output of this research is Eczema Care Online for Families, an online intervention for parents/carers of children with eczema, which is being evaluated in a randomised trial.

## INTRODUCTION

Eczema affects around one in five children.^1^ National Institute for Health and Care Excellence (NICE) guidelines recommend daily use of emollients, plus topical corticosteroids (TCS) to treat eczema flare-ups.^1^ The main cause of treatment failure is underuse of topical treatments, which can reduce quality of life and increase healthcare costs.^1^ Underuse is often owing to practical and psychosocial barriers, such as treatment being time-consuming, concerns about TCS, and child resistance.^2^^–^^5^ Little is known about barriers/facilitators to parents/carers managing and co-managing eczema with older children.^4^

The majority of eczema self-management interventions involve face-to-face education, which can be effective, but their cost-effectiveness is unknown, and uptake can be poor.^6^^,^^7^ Previous digital interventions targeting parents/carers typically focus on young children,^8^ or include multiple components (for example, education, online monitoring, and face-to-face consultation).^9^ Other interventions include written action plans but effectiveness has not yet been evaluated.^10^ Systematic reviews show the need for systematically developed evidence-based interventions to support self-management of eczema.^9^^,^^11^^,^^12^

Key to developing acceptable, feasible, and ultimately effective interventions is understanding the needs and context of target users.^13^^,^^14^ The current research aimed to systematically develop an online behavioural intervention for parents/carers of children aged 0–12 years with mild to severe eczema (‘Eczema Care Online [ECO] for Families’). It aims to support parental management and parent/child co-management of eczema. Intervention development was informed by qualitative studies that explored parents’/carers’: 1) barriers and facilitators to managing and co-managing eczema; and 2) views of the online intervention, its acceptability, and its feasibility. This article describes the research underpinning the development of ECO for Families, which was developed in line with Medical Research Council guidance on developing and evaluating complex interventions.^15^^,^^16^

**Table:** How this fits in

Parents/carers report multiple barriers to managing childhood eczema, including limited information about eczema, its treatments, and child resistance, which could potentially be addressed through online interventions. This article identifies key issues/challenges for families managing childhood eczema and solutions to consider when supporting them, and describes the development of an online intervention to support families (‘Eczema Care Online for Families’). A key finding of this research is that even parents/carers with extensive experience of looking after childhood eczema have gaps in knowledge around treatment, which healthcare professionals could help identify and address, particularly around why, when, and how to use emollients and topical corticosteroids (TCS). The article introduces terminology to help clarify the purpose of emollients (moisturising creams) and TCS (flare control creams), and reflect parents’/carers’ language.

## METHOD AND RESULTS

ECO for Families was developed during 2017–2019 using a theory-, evidence-, and person-based approach (PBA),^13^^,^^17^^,^^18^ which grounded intervention development in an in-depth understanding of target users’ needs, challenges, and context (that is, parents/carers of children aged 0–12 years with mild to severe eczema). Five behaviours were targeted, which were agreed through stakeholder consultation as being crucial to effective eczema care:

- reactive application of TCS to get control of skin inflammation;
- increased use of emollients to keep control of skin inflammation;
- improved management of irritants/triggers;
- reduced scratching (children); and
- improved emotional distress management (children).

Stakeholder consultation throughout intervention development ensured that advice in the intervention was medically correct, evidence based, and presented in an acceptable and accessible way.^19^ The intervention development group, which included the intervention development team and stakeholders, comprised 16 experts in dermatology and intervention development: two consultant dermatologists, a nurse consultant, a professor of nursing, two skin researchers, three GPs, three health psychologists, and four research psychologists. The group also included two patient representatives: two mothers of children with eczema, one of whom had eczema herself and was a patient advocate in eczema. For further detail of how patient and public involvement was used to complement the PBA in ECO for Families see Muller *et al*.^20^

**Figure 1.**
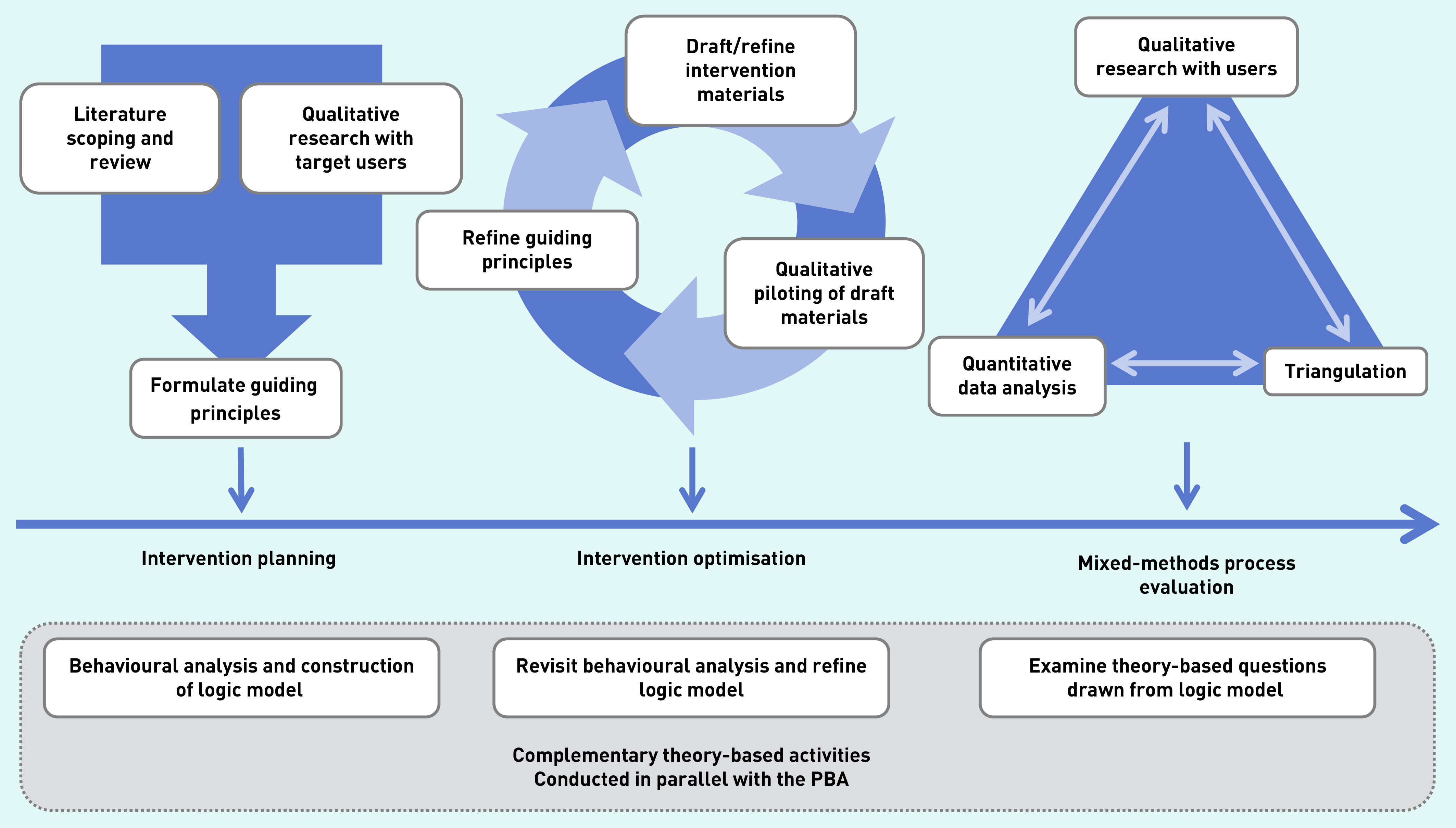
*Overview of the person-based approach (PBA) to intervention development.* *Source: reproduced with permission from https://www.lifeguideonline.org/pba.*

In keeping with the first two steps of the PBA (Figure 1), ECO for Families was developed in two iterative stages: 1) intervention planning; and 2) intervention optimisation. Evidence of potential barriers and facilitators to the behaviours targeted by the intervention, and key factors affecting how families managed and co-managed their child’s eczema, were explored. The methods and findings at each stage are set out, along with how this evidence was used to plan, develop, and optimise the intervention. As per previous intervention development papers using the PBA,^17^^,^^21^^,^^22^ the methods and results are presented together by stage to illustrate how these informed intervention planning and optimisation.

### Stage 1: Intervention planning

Stage 1 focused on intervention planning (Figure 1). In ECO, this process was informed by two key studies: a systematic review/qualitative synthesis of the literature,^4^ and interviews with parents/carers. Evidence from these studies was used to develop three outputs:

- ‘guiding principles’ to inform intervention design — these specified intervention objectives and features for maximising families’ engagement with the intervention (Box 1);^13^^,^^17^^,^^18^
- a behavioural analysis identifying barriers/facilitators to the five target behaviours and potential intervention components and techniques that would address them — intervention components and techniques were then mapped to theory using a behaviour change framework to ensure no important behaviour change techniques were missed and to systematically describe the intervention (Supplementary Table S1); and^23^^,^^24^
- a logic model — this outlined key intervention components and techniques, and hypothesised mechanisms of action and target outcomes.

**Box 1.** Guiding principles for intervention design

**Issues**	**Source**	**Intervention design objectives to address issues**	**Key features to address issues**
Parents/carers may not have a lot of time; eczema treatment can be time consuming and may be challenging to fit into their daily routine	SR, EO	To create an intervention that is engaging and easy to navigate, in which parents/carers can quickly find the relevant information	Make most intervention content optional so it can be accessed when/if it is needed Add filtering questions to help signpost parents/carers to relevant modules Use a modular layout so that parents/carers can quickly identify and select relevant topics Ensure information is concise, presented in short chunks Provide information in a range of formats to improve accessibility (for example, audio-visual features, interactive features)
Parents/carers may feel distressed by the impact eczema has on their child. They may be struggling to manage their child’s eczema, may be sleep deprived, or may worry about the long-term impact of eczema on their child. They may also feel distressed by their child’s reaction to treatments (for example, if the child finds it uncomfortable or painful), which may lead them to avoid, delay, stop, or use treatments less often than needed.	PPI, SR, I	To reduce parents’/carers’ feelings of helplessness, frustration, self-blame, and guilt about their child’s eczema	Validate and normalise parents’/carers’ feelings around eczema and its management Emphasise things that parents/carers can do to help manage their child’s eczema, including tips and quotes from other parents/carers Acknowledge that there are precipitating factors that are out of their control and identify what parents/carers can do to manage flare-ups Avoid messages that may be viewed as blaming parents/carers for eczema flare-ups Provide emotional management techniques that can help parents/carers manage difficult emotions
Young children may resist treatments because they dislike them and may not understand why they need them. As children get older, they increasingly encounter situations where they need to take more responsibility for managing their eczema (for example, starting school, socialising outside the home). They may also want to start to self-manage, so will need to develop their own knowledge and skills for managing eczema.	PPI, SR, I	To facilitate co-management of eczema between parents/carers and their child to support their child’s treatment adherence, and support their child’s transition towards self-management	Provide suggestions for ways parents/carers can involve their child in managing their treatment Provide age-appropriate materials to help children learn about eczema and its management
Children may find eczema painful, itchy, unpleasant, or distressing. They may not understand what eczema is, or why they need to do the things that help them manage their eczema. They may find topical treatments painful, unpleasant, frustrating, or boring, which may lead them to avoid using treatments or use them less than is needed.	PPI, SR, I	To reduce children’s feelings of distress, anxiety, hopelessness, and frustration around eczema and its treatment	Help parents/carers to understand children’s feelings Provide age-appropriate tools/activities to help children manage difficult emotions related to eczema and its treatment to use on their own or with parents/carers Provide age-appropriate explanations about eczema and its treatments to help children make sense of eczema and its treatment

*EO = expert opinion. I = stage 1 interviews. PPI = patient–public involvement representatives. SR = systematic review.*

These key studies and their intervention development outcomes are described below.

### Systematic review/qualitative synthesis of research on families’ views and experiences of managing eczema

#### Method

A systematic review/thematic synthesis examined patients’ and parents’/carers’ views and experiences of eczema, eczema treatments, and barriers/facilitators to managing eczema, reported in full elsewhere.^4^ This review identified 39 papers from 32 studies.^25^

#### Results

Parents/carers reported substantial physical and emotional impact of caring for their child’s eczema, including feeling exhausted, guilty, and worried. They reported needing to change behaviours and routines to adapt to managing eczema. Key barriers included:

- lack of information about eczema and how to use treatments;
- receiving negative or conflicting advice about topical treatments (particularly TCS);
- treatment being time intensive and burdensome, particularly applying topical treatments, and managing irritants/triggers;
- parents/carers and children disliking topical treatments (owing to feel/smell/stinging);
- child resistance to parents applying topical treatments;
- concerns about the safety of TCS and ‘unnatural’ ingredients included in emollients;
- uncertainty about how and when to use TCS; and
- doubts about the effectiveness of topical treatments.

### Interviews

#### Method

Participants were recruited through mail-outs from 16 GP surgeries and opportunistic recruitment in three NHS hospitals. Participants needed to have a child aged 0–12 years with diagnosed eczema who had one or more eczema prescriptions in the previous 12 months, and to be able to communicate in English. Participants received an invitation pack, including an information sheet, and a reply slip to express interest in the study. To gather a diverse range of views and cover a range of developmental stages, participants were purposively sampled on the child’s age, sex, eczema severity, and geographical location. Selected participants were invited to a face-to-face, semi-structured interview and consented before the interview. All interviews were conducted at participants’ homes between March and July 2018 by a female research psychologist experienced in qualitative research. Interviews explored parents’/carers’ views and experiences of managing and co-managing their child’s eczema, treatment barriers/facilitators, and terminology used for topical treatments (see Supplementary Box S1 for topic guide). Interviewees received a £10 voucher. Recruitment continued until saturation was reached for main themes.

Interviews lasted 45–60 minutes, were audiorecorded, transcribed verbatim, and analysed using inductive thematic analysis.^26^^,^^27^ Data were managed in NVivo 12. A coding manual was created with audit trail. Constant comparison between transcripts, codes, and themes ensured coherency, and that diverse cases were identified. Analysis was iterative, with codes and themes updated following team discussion and stakeholder consultation. This process facilitated a richer and more nuanced understanding of the data by including the perspectives of dermatology and non-dermatology specialists and people with and without lived experience.

As the focus was on understanding parents’/carers’ experiences of managing/co-managing eczema with their child, the analysis focused predominantly on barriers and facilitators to eczema management to inform the intervention. Results presented here focus on novel findings that extend those in the systematic review.

#### Results

Thirty parents/carers (all female) were interviewed. The majority of interviewees (*n* = 28; 93%) were recruited through primary care. See Table 1 for child characteristics.

**Table 1.** Characteristics of children of parents/carers taking part in the interviews

**Child characteristics**	**Stage 1: Interviews (*N* = 30)**		**Stage 2: Think-aloud interviews (*N* = 25)**	
	
	***n***	**%**	***n***	**%**
Age group				
• Infant (<1 year)	3	10	1	4
• Toddler (1–2 years)	7	23	5	20
• Preschool (3–5 years)	5	17	10	40
• Younger school age (6–8 years)	7	23	3	12
• Older school age (9–12 years)	8	27	6	24

Sex				
• Female	15	50	16	64
• Male	15	50	9	36

Eczema severity^a^				
• Mild	14	47	13	52
• Moderate	10	33	10	40
• Severe	6	20	2	8

a *Participants self-reported what they thought their child’s eczema severity was (mild, moderate, severe).*

Parents/carers typically referred to topical treatments as ‘creams’, irrespective of their specific type (gel/ointment/lotion/cream). Emollients were generally called just ‘creams’, ‘emollient creams’, or by their brand name. Only a few participants called them ‘emollients’ or ‘moisturisers’. TCS were usually called ‘steroid creams’, ‘steroids’, or occasionally by product name. One parent/carer called them ‘strong creams’ when discussing them with her child.

Thematic analysis identified six key themes, listed below:

a) Incomplete knowledge about eczema and its treatments: most parents/carers described having received little information explaining eczema and its treatments. Several wanted more information, particularly hints and tips from other parents/carers. Several were unsure what caused eczema flare-ups. A couple wondered how puberty might affect eczema:

‘*When her skin is changing due to teenage-hood … when she wants to start using make-up; does it interfere with eczema?’*

(Parent/carer 29, 12-year-old daughter, moderate eczema)

A few parents/carers said they were not sure how best to use emollients, particularly whether they needed to be used regularly. One was unsure if they used them too much:

*‘I stopped using* [emollient] *because it didn’t seem to flare up … if I’d kept using it all winter, would she not have flared up now? Or would she have flared up anyway … ?’*

(Parent/carer 9, 18-month-old daughter, mild eczema)

A few discussed previously using TCS incorrectly or being uncertain about when or how to use them:

*‘I didn’t know how much* [TCS] *to put on … I didn’t know what was right and wrong.’*

(Parent/carer 5, 2-year-old daughter, moderate eczema)

b) Concerns and doubts about the safety and effectiveness of TCS and emollients: most parents/carers had concerns about TCS. Some described them as a necessary evil; they did not like them but knew they worked. Some worried about skin thinning. A couple tried to avoid using them and described this as a dilemma:

*‘It was a constant battle between trying not to use them* [TCS] *and having to use them … Do we need* [TCS] *or can we get a grip of this without?’*

(Parent/carer 26, 6-year-old daughter, moderate eczema)

Most parents/carers felt that emollients were effective in reducing itch and keeping control of eczema. However, a few were unconvinced or thought they worked less well over time:

*‘It seems after a certain amount of time* [emollients] *… lose their magic.’*

(Parent/carer 28, 5-year-old daughter, severe eczema)

c) Process of trial and error: most parents/carers described a process of trial and error to work out how best to manage their child’s eczema, which might relate to incomplete knowledge around eczema management. This process included: finding the right emollient, developing an emollient routine, and making changes to manage irritants and scratching. A few parents/carers described adapting their regimen over time as their child changed:

*‘I have to think … what’s caused this* [flare-up] *to come on? … I just try and work it out as we go along.’*

(Parent/carer 15, 6-year-old son, mild eczema)

Several expressed a desire to carry on as usual and to find a balance between managing their child’s eczema and leading a ‘normal’ life:

*‘If we have a flare we’ll just deal with it at the end of the day … I’m not going to stop her from having a childhood …’*

(Parent/carer 8, 8-year-old daughter, severe eczema)

d) Negative impact of eczema and its treatments on parents: most parents/carers described treatments as time consuming, unpleasant, and messy. Some felt exhausted owing to treatment burden and sleep deprivation, distressed at seeing their child upset or in pain, or felt they lacked control over their child’s eczema:

*‘I feel heartbroken, especially when I see their skin quite bad … you feel helpless.’*

(Parent/carer 1, 5-year-old son, mild eczema)

e) Child acceptance and rejection of topical treatments: several parents/carers discussed their child disliking topical treatments. Several felt toddlers were more challenging as they would scream or run away, but some described arguments with older children over delaying or avoiding emollients, and children getting annoyed at parent/carers’ prompting them. However, most felt that, if their child understood why topical treatments were needed, it helped their child to accept them, and that age-appropriate materials explaining eczema and its treatments would be helpful:

*‘Books or something like that would be good, or anything … interactive. Colouring on how to apply cream … something that helps them be more educated on what they can do.’*

(Parent/carer 12, 6-year-old son, mild eczema)

Several felt that examples of other children talking about eczema and how they self-manage might help normalise eczema for their child:

*‘Videos or clips from other kids who’ve got it … then they feel … they’re in the same boat, that other people are experiencing that.’*

(Parent/carer 29, 12-year-old daughter, moderate eczema)

Some felt that establishing a routine helped normalise emollients into their child’s day. Others talked about making treatment times more enjoyable with toys or rewards.

f) Reluctance to transition to parent–child co-management and child self-management: some parents/carers talked about letting younger children put their emollients on; however, there were mixed experiences around this. For some it was a deliberate move to teach their child about their emollients. Others described it as a mistake because of the resulting mess:

*‘I’m quite reluctant to … “No, let me do it.” Then you’ve got the handprints all over the mirror and you just find sticky stuff everywhere.’*

(Parent/carer 10, 5-year-old daughter, moderate eczema)

Although some parents/carers had positive views of transitioning care to their child, these tended to be older or ‘very mature’ children. Several felt their child was too young. Some had difficulties letting go of their child’s care as they felt that their child would not look after their eczema properly because they were not physically capable, or they were not motivated. Some parents/carers managed this by prompting their child or helping them when needed. However, this reluctance to hand over care was apparent even in children close to adolescence who wanted to self-manage:

*‘I would prefer to be doing it* [emollients] *… because I know that it’ll be getting done properly … she’s going through puberty, her body’s changing, she doesn’t want me coming in … there’s been a few arguments, because, sometimes, I don’t think she’s looking after herself properly.’*

(Parent/carer 6, 12-year-old daughter, severe eczema)

Only a few parents/carers talked about TCS in the context of co-management; most felt concerned about letting their child apply TCS or felt they needed to be older, reflecting their concerns about TCS:

*‘I don’t let her do steroid cream by herself … I’m very conscious that I don’t want her putting too much on.’*

(Parent/carer 17, 9-year-old daughter, mild eczema)

Some parents/carers also doubted that other adults would apply emollients as effectively/consistently as they would. A few doubted school would manage eczema or had had schools refuse to manage eczema. Some reported their child feeling uncomfortable using emollients at school. Although a couple of parents/carers described positive experiences at school, this was when there were close links with the school through a school nurse or the parent working there, facilitating eczema management.

### Intervention development

**Figure 2.**
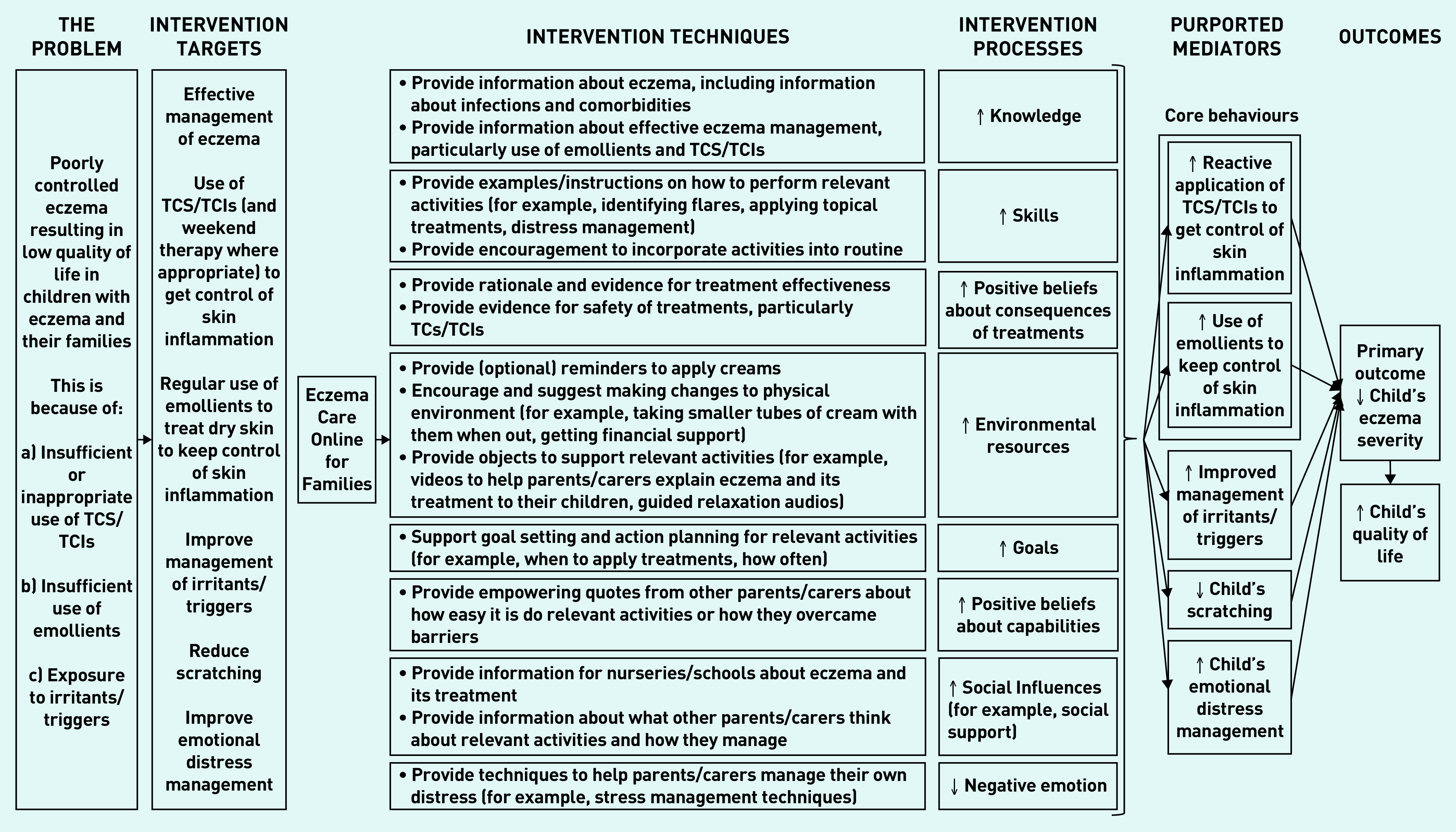
*Final intervention logic model outlining key components and hypothesised mechanisms of Eczema Care Online for Families.* *TCIs = topical calcineurin inhibitors. TCS = topical corticosteroids.*

Key issues influencing intervention design and barriers/facilitators to intervention target behaviours were extracted from the systematic review and interviews, and used to inform the guiding principles and behavioural analysis. Key barriers are summarised in Box 1 and Box 2, along with the intervention features/ingredients for addressing them (see Supplementary Table S1 for the full behavioural analysis). An intervention logic model based on the behavioural analysis can be found in Figure 2, outlining key components, techniques, hypothesised mechanisms of action, and target outcomes.^28^

**Box 2.** Summary of key barriers and intervention ingredients

**Key barriers to target behaviours**	**Source**	**Key domain targeted**	**Intervention ingredients to address key barriers**
Incomplete knowledge about eczema, its triggers, and its treatment	SR, I, TA	↑Knowledge about eczema and its management	*Parents/carers* Provide information about eczema, its treatment, and triggers Provide advice about identifying when emollients/TCS are needed for a range of different skin types and severities Provide information on when to apply emollients/TCS and when they should use them, and for how long, including advice on identifying the start and end of eczema flare-ups Provide information about how emollients and TCS differ in terms of their function and how they should be used together Provide instructional video/photos of how to correctly apply TCS *Children* Explain what eczema is and how it is treated in simple language using videos
Limited skills for managing and co-managing eczema and its treatments (for example, using creams, supporting transition to child self-management)	SR, I, TA	↑Skills to manage and co-manage eczema with their child	*Parents/carers* Use videos to demonstrate how emollients should be applied and how much Provide suggestions for activities around emollient use to make emollient times more fun and interesting for children (for example, imaginary games, singing, special toys for emollient times) Encourage parents/carers to involve their child in applying emollients so they can learn how to do it themselves *Children* Use videos to demonstrate how emollients should be applied and how much
Concerns and doubts about emollients and, especially, TCS	SR, I	↑Positive beliefs about consequences (of using emollients and TCS)	*Parents/carers* Provide a rationale for how emollients and TCS help to manage eczema including when eczema is only mild or not visible on the skin Provide persuasive and credible information about the effectiveness of emollients and TCS, including scientific evidence, user stories, quotes, and videos Provide advice about trying out new emollients and finding an emollient that works, including advice on when an emollient should be abandoned to try a new one Encourage use of a 2-week challenge to evaluate how regular use of an emollient improves eczema symptoms (redness, soreness, itching), and prompt trying a different emollient if it does not Provide advice on how to support the child to tolerate the treatments better (for example, distraction, relaxation) Provide user stories/quotes about how they dealt with unpleasant reactions in their child Acknowledge that the process of finding the right emollient can be frustrating/overwhelming/disheartening Reassure parents/carers that it is OK to ask to change emollients if their child cannot tolerate their current emollient *Children* Explain how emollients and TCS help eczema using easy-to-understand videos

*EO = expert opinion. I = stage 1 interviews. PPI = patient-public involvement representatives. SR = systematic review. TA = stage 2 think-aloud interviews. TCS = topical corticosteroids.*

Initial intervention content was written in Microsoft Word informed by the guiding principles and behavioural analysis. It was shared with stakeholders to ensure medical accuracy and obtain patient/parent feedback on acceptability and feasibility before think-aloud interviews in Stage 2. This content was then developed into a website using LifeGuide software, and thoroughly tested to ensure functionality across different types of devices (computers, mobile phones, tablets).

A modular intervention was developed with modules focusing on topics related to eczema including treatment use and psychosocial issues (for example, stress). Parents/carers were initially guided through a short introductory module, which had three key purposes:

- establish credibility of the intervention;
- explain eczema and the skin barrier; and
- briefly explain key treatments (emollients and TCS) and how to use them.

This aimed to ensure parents/carers had the basic knowledge/skills for managing eczema.

A key aspect of the intervention was the terminology developed for describing emollients, which were called ‘moisturising creams’, and TCS, which were called ‘flare control creams’. This was done to reflect parents’/carers’ own terminology (that is, ‘creams’) and to help make clear their different purposes, particularly the role of TCS and topical calcineurin inhibitors (TCIs) in treating eczema flare-ups.

**Figure 3.**
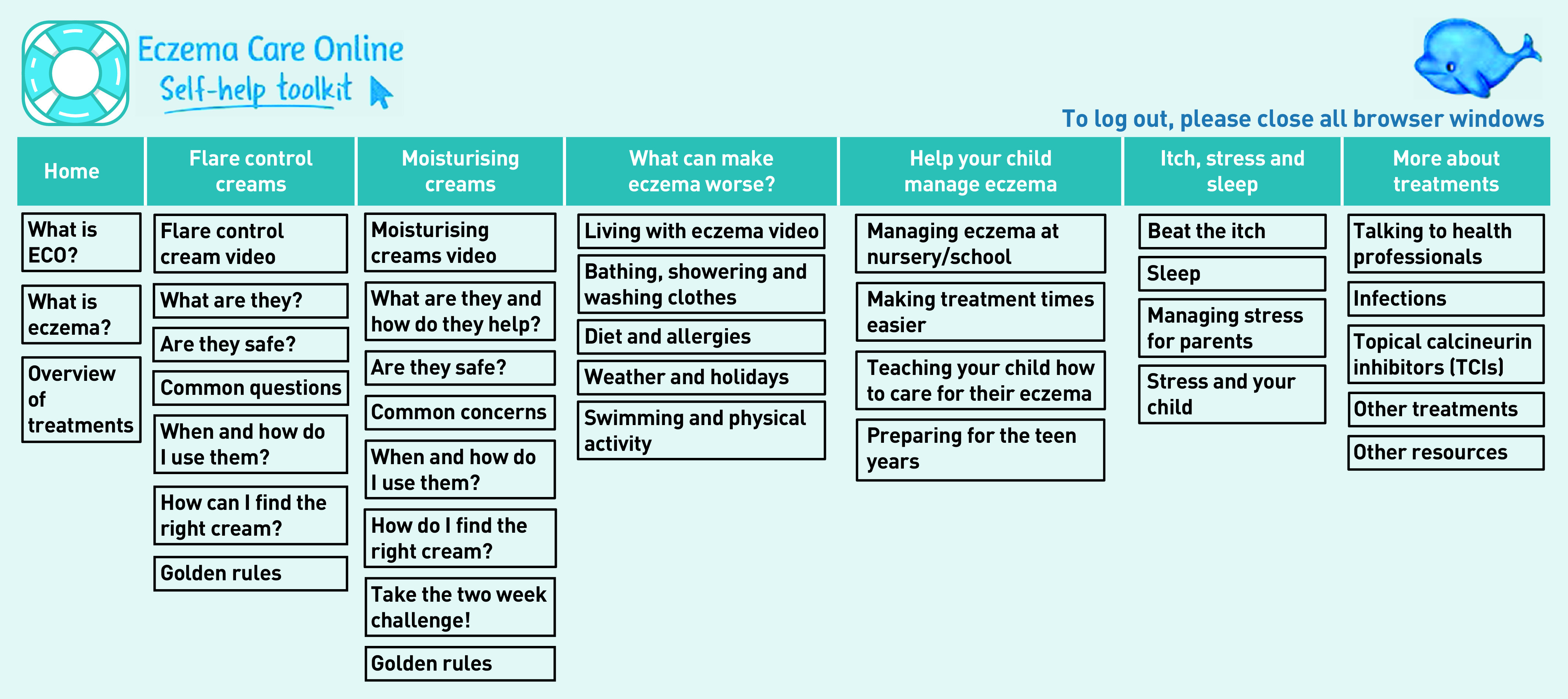
*Overview of modules and quick access menus in Eczema Care Online for Families. ECO = Eczema Care Online.*

At the end of the introduction, parents/carers could take a brief quiz to assess their child’s eczema, the results of which then recommended one of two core modules: ‘getting control using flare control creams’ or ‘keeping control using moisturising creams’. These provided more information about treatments, addressed common concerns, and provided information and photos/video demonstrations of how best to use treatments. Additional modules were provided through drop-down menus (Figure 3) to allow parents/carers to access a range of topics, including: managing irritants and triggers (‘what can make eczema worse’), co-management (‘help your child manage eczema’), managing the impact of eczema (‘itch, stress, and sleep’), and other treatments and related issues (‘more about treatments’).

A full description of the intervention was compiled using TIDieR (Template for Intervention Description and Replication) guidance for reporting intervention development (Supplementary Box S2).^29^

### Stage 2: Intervention optimisation Think-aloud Interviews

Stage 2 focused on optimising the prototype of the digital intervention using iterative think-aloud interviews with parents/carers. These aimed to elicit feedback on the prototype to inform refining its content and design so that it was more acceptable and feasible for parents/carers to follow. The new terminology around ‘moisturising creams’ and ‘flare control creams’ was also assessed.

#### Method

Face-to-face think-aloud interviews^30^ were conducted between October 2018 and April 2019 by a female research psychologist experienced in qualitative research and a medical student (supervised by the psychologist). Participants were recruited using mail-outs from eight GP surgeries using the same eligibility criteria and purposive sampling approach as Stage 1. Recruitment continued until saturation was reached. Interviewees received a £10 voucher. Interviews lasted 45–90 minutes, used a standard think-aloud interview approach involving minimal prompting to elicit participants’ reactions, and were conducted at the participant’s homes. Participants read sections of the website and said aloud their immediate reactions to the content (see Supplementary Box S3 for topic guide). Interviews were audiorecorded and transcribed verbatim. Data were analysed concurrently to the interviews using a Table of Changes,^31^ in which all positive and negative comments were collated, and potential changes identified and prioritised in terms of feasibility and importance of changes in increasing acceptability and feasibility of the intervention. Minor changes to the intervention were agreed within the intervention development team with key issues discussed with stakeholders to support reflexivity and medical accuracy of modified intervention content. Interviews were carried out iteratively, with feedback from earlier interviews informing modifications to optimise the intervention, and later interviews using revised prototype interventions to seek feedback on modifications.

#### Results

Twenty-five parents/carers (*n* = 23 female; 92%) were interviewed. See Table 1 for child characteristics.

Views of the prototype were generally positive: particularly the new terminology for eczema treatments, the wide variety of topics covered, and the videos, parent/carer quotes, and tips. However, participants felt the content was lengthy and repetitive, and wanted quicker access to main modules. A key issue was that many parents/carers initially felt the content was not relevant to them if they had been looking after their child’s eczema for a while. Despite this, when going through the content participants still identified things that they had not known, such as why and how emollients help keep eczema under control and how to correctly apply treatments (for example, using TCS until 2 days after the eczema flare-up clears, applying topical treatments in the direction of hair growth). Parents/carers also felt they had gained useful practical tips they had never tried before, such as putting creams in the fridge to make them cool to sooth itching or setting reminders on phones:

*‘I’ve learnt so much this morning that I didn’t know about eczema, and I thought I knew quite a lot!’*

(Parent/carer 16, 2-year-old daughter with mild eczema)

### Intervention optimisation

Modules were streamlined and made more interactive to increase user choice and autonomy using optional click-outs and pop-ups. In particular, the core content in the introductory module was cut from 21 to nine short pages. Readability was improved on individual pages by: 1) highlighting key messages using bold text; 2) using bullet points; and 3) separating text using boxes. Signposting, quotes, and tips were added to the introductory module and first page of the core modules to emphasise that:

- the website provided up-to-date information about eczema and its treatments;
- core modules would be basic at the start but then progress; and
- even parents/carers who had been caring for their child’s eczema for a while had learnt new things.

## DISCUSSION

### Summary

This article describes the intervention development of ECO for Families, an online behavioural intervention that supports parents/carers of children aged 0–12 years to manage and co-manage their child’s eczema. A previous systematic review/qualitative synthesis of the literature and interviews helped to identify key barriers that needed to be addressed, including:

- incomplete knowledge about eczema, its triggers, and its treatment;
- concerns and doubts about emollients and, especially, TCS; and
- limited skills for managing and co-managing eczema and its treatments.

This information was used to identify appropriate behaviour change techniques and develop a prototype intervention. Think-aloud interviews were used to optimise the intervention.

### Strengths and limitations

A strength of ECO for Families is the use of a theory-, evidence- and person-based approach, which ensured development was systematic and informed by in-depth qualitative research and theory.^9^ A limitation of the research underpinning ECO for Families was that there were few interviews with parents/carers of children with severe eczema aged under 1 year, particularly in the think-aloud interviews. Most parents/carers were female, so little is known about the views of fathers/male carers. However, there was diversity in child ages, sex, and eczema severities. Interviews were also carried out and analysed by the intervention development team, which may have led to bias. Nonetheless, stakeholder consultation with a wider range of stakeholders, including patient/parent/carer representatives, was used throughout to ensure key aspects were not missed.

### Comparison with existing literature

Existing literature supports the finding that parents/carers feel there is a lack of reliable information about eczema and its treatments,^32^^,^^33^ and that most parents/carers navigate this through a process of trial and error.^5^ The finding that parents/carers report doubts and concerns about topical treatments, particularly TCS, is also supported.^2^^,^^3^

To the authors’ knowledge, there has been no research into parents’/carers’ views and experiences of co-managing eczema with children,^4^ although research with children suggests conflict between parents/carers and children.^34^ The current research suggests that parents/carers may be reluctant to transition care to their child, even when children want to self-manage, which is an issue that has been identified in other long-term health conditions, such as asthma and diabetes.^35^ The current intervention supports co-management and provides resources for parents/carers to teach their child about eczema and its treatments. Qualitative research suggests that young people with eczema struggle to make sense of eczema and its treatments despite having it since childhood.^34^ This research highlights the importance of information given about eczema and its treatments in childhood, and the potential impact this can have on treatment use. It is hoped that the focus on co-management in ECO for Families may help parents/carers support their child’s transition to self-management as they get older.

### Implications for research and practice

A key finding in the think-aloud interviews was that parents/carers who have been caring for their child’s eczema for a while may believe that they already know how to use emollients and TCS. However, it was clear that there were still important gaps in their knowledge. This supports NICE recommendations that healthcare professionals refresh parents’/carers’ knowledge of how to use treatments when they reconsult.^1^ The current research suggests that key elements include when and how to use emollients and TCS, and, crucially, their different purposes. Parents’/carers’ skills can be enhanced by demonstrating how to use treatments, and involving children in this could help with transitioning to co-management. Behaviour change strategies for addressing concerns and doubts are also suggested, which include detailed explanation of when and how to use TCS safely.

In conclusion, ECO for Families aims to address key barriers to parents/carers effectively managing their child’s eczema. It supports a co-management approach, with the aim of facilitating the child’s later transition to self-management. Effectiveness and cost-effectiveness of ECO for Families are being evaluated in a randomised controlled trial,^36^ alongside a nested process evaluation to explore parents’/carers’ experiences of the intervention, factors influencing user engagement and outcomes, potential mechanisms of actions, and issues for implementation. A similar intervention targeting young people with eczema has also been developed,^37^ and is currently being trialled.^36^
